# Exploring the JAK/STAT Signaling Pathway in Hepatocellular Carcinoma: Unraveling Signaling Complexity and Therapeutic Implications

**DOI:** 10.3390/ijms241813764

**Published:** 2023-09-06

**Authors:** Hyunjung Park, Sangjik Lee, Jaehun Lee, Hyuk Moon, Simon Weonsang Ro

**Affiliations:** Department of Genetics and Biotechnology, College of Life Sciences, Kyung Hee University, Yongin-si 17104, Republic of Korea; molly921@khu.ac.kr (H.P.); steven960@khu.ac.kr (S.L.); hjjk00000@khu.ac.kr (J.L.); hmoon@khu.ac.kr (H.M.)

**Keywords:** hepatocellular carcinoma, JAK/STAT signaling, cytokine, targeted therapy

## Abstract

Hepatocellular Carcinoma (HCC) continues to pose a substantial global health challenge due to its high incidence and limited therapeutic options. In recent years, the Janus Kinase (JAK) and Signal Transducer and Activator of Transcription (STAT) pathway has emerged as a critical signaling cascade in HCC pathogenesis. The review commences with an overview of the JAK/STAT pathway, delving into the dynamic interplay between the JAK/STAT pathway and its numerous upstream activators, such as cytokines and growth factors enriched in pathogenic livers afflicted with chronic inflammation and cirrhosis. This paper also elucidates how the persistent activation of JAK/STAT signaling leads to diverse oncogenic processes during hepatocarcinogenesis, including uncontrolled cell proliferation, evasion of apoptosis, and immune escape. In the context of therapeutic implications, this review summarizes recent advancements in targeting the JAK/STAT pathway for HCC treatment. Preclinical and clinical studies investigating inhibitors and modulators of JAK/STAT signaling are discussed, highlighting their potential in suppressing the deadly disease. The insights presented herein underscore the necessity for continued research into targeting the JAK/STAT signaling pathway as a promising avenue for HCC therapy.

## 1. Introduction

Hepatocellular Carcinoma (HCC) stands as the predominant primary liver malignancy, constituting approximately 85% to 90% of all cases. It continues to be a substantial global health concern due to its increasing incidence and the limited therapeutic options available [1,2,3]. According to data from the Global Cancer Statistics (GLOBOCAN 2020, available at https://gco.iarc.fr (accessed on 1 August 2021), there are 906,000 new cases of liver cancer and 830,000 liver cancer-related fatalities annually worldwide [4]. The highest incidence and mortality rates of HCC are notably concentrated in East Asia, although there is a mounting trend of HCC incidence and mortality in various regions of Europe and the United States. The data further delineate regional disparities, with approximately 657,000 cases occurring in Asia, with more than half of them arising in China. Europe registers 90,000 cases, while Africa records 70,000, North America 50,000, Latin America and the Caribbean 40,000, and Oceania 5000 cases annually. The annual mortalities due to HCC likewise follow a similar geographical distribution, with the highest figures seen in Eastern Asia and Europe, accounting for 669,000 and 79,000 deaths, respectively. This is followed by Africa (67,000), Latin America and the Caribbean (38,000), North America (35,000), and Oceania (4000). In terms of 5-year prevalence data, the pattern remains consistent, with the majority of patients, approximately 74% out of a total of 995,000, residing in Asia, followed by Europe at 8.6%, Africa at 8.4%, North America at 5%, Latin America and the Caribbean at 4%, and Oceania at 0.4%.

In the context of therapeutic interventions for HCC, surgical resection and local ablation therapy are preferred for early-stage HCC. Nonetheless, the incidence of recurrence within a five-year period following such therapeutic interventions can be as high as 70% [1,3]. In the case of advanced HCC, the recommended treatment option is the systemic molecular target therapy [1,2]. Within this realm, an array of small-molecule compounds has undergone evaluation in patients afflicted with HCC, with a particular focus on targeting receptor tyrosine kinases (RTKs) [5]. As of current knowledge, however, the clinical outcomes from RTK-targeting molecular therapies have yielded results that are less than entirely satisfactory. A prominent example is sorafenib, which stands as the foremost compound among RTK inhibitors currently administered to HCC patients; nevertheless, its therapeutic benefits for individuals with HCC have shown limitations [6,7].

Given the profound diversities intrinsic to the molecular landscape of HCC and the formidable challenges entailed in devising universally effective therapeutic strategies for this disease, the identification of molecular signaling pathways perturbed in HCC assumes fundamental importance in developing therapeutic targets as a personalized medical approach [8,9]. In the majority of HCC cases, the presence of chronic hepatic inflammation and cirrhosis is concomitant, often induced by diverse etiological factors such as chronic hepatitis B virus (HBV) or hepatitis C virus (HCV) infections, alcohol abuse, diabetes, obesity, and hepatic lipid accumulation, among others [1,2]. In a chronically injured liver, there is an aberrant augmentation of inflammatory cells and inflammatory responses, which, in turn, fosters a tumorigenic microenvironment and activates various oncogenic molecular signaling pathways [10,11].

A multitude of pro-inflammatory cytokines, including various interleukins, growth factors, and interferons, are elevated in livers with chronic inflammation [10,12], favoring the activation of the Janus Kinase (JAK) and Signal Transducer and Activator of Transcription (STAT) pathway, commonly referred to as the JAK/STAT pathway. Extracellular signals from a plethora of cytokines are translated via the JAK/STAT pathway into intracellular responses governing various cellular processes, such as proliferation, differentiation, apoptosis, and immune response [13,14]. In the context of HCC, the aberrant activation of the JAK/STAT pathway has garnered significant attention due to its profound impact on tumorigenesis, metastasis, angiogenesis, and immune evasion [15].

This review paper aims to provide a comprehensive overview of the current state of knowledge regarding the role of the JAK/STAT pathway in HCC. We will explore the implications of JAK/STAT pathway dysregulation in HCC, which is triggered by a variety of cytokines. Additionally, we will discuss the results from recent preclinical and clinical studies that target the JAK/STAT signaling cascade as a potential therapeutic approach for treating HCC.

## 2. JAK/STAT Signaling Pathway

### 2.1. Overview of JAK/STAT Signaling

The JAK/STAT pathway is a vital cellular cascade that controls a wide range of processes, including cell proliferation, differentiation, and apoptosis [16,17]. Its significance is particularly pronounced in modulating immune and inflammatory responses [18,19]. Additionally, it plays a role in hematopoiesis and the functioning of hematopoietic stem cells (HSCs) [20,21,22]. Notably, the JAK/STAT pathway contributes to diverse liver functions, such as hepatic proliferation, regeneration, hepatoprotection, and metabolic processes such as gluconeogenesis [23,24]. This signaling pathway also plays a crucial role in various stages of tumorigenesis, including epithelial–mesenchymal transition (EMT) and metastasis. Moreover, it is highly associated with the generation and maintenance of cancer stem cells (CSCs), which play pivotal roles in therapy resistance and metastasis [25,26].

**Figure 1:**
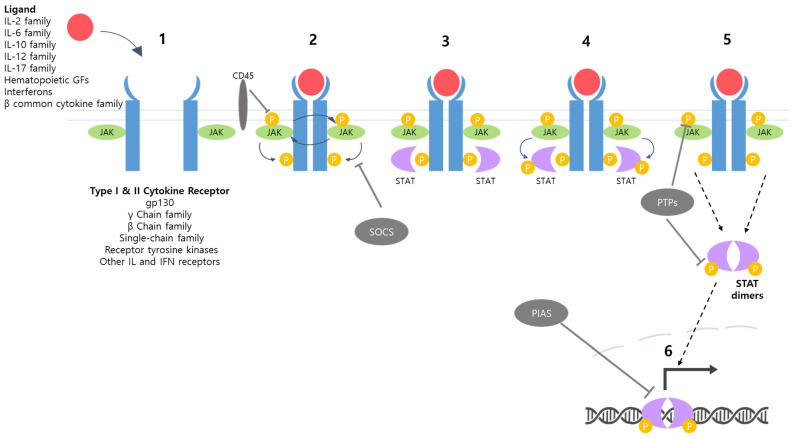
Schematic illustration of the JAK/STAT signaling pathway. Ligand binding to cytokine receptor leads to the formation of receptor dimerization (1). The dimerization induces the transphosphorylation of JAK as well as phosphorylation of the cytoplasmic tails of the receptors (2). STAT binds to the phosphorylated site of the receptor (3). Subsequently, JAK phosphorylates STAT recruited to the receptors (4). Phosphorylated STATs dissociate from the receptors and form a dimer (5). STAT dimers translocate into the nucleus and induce the transcription of target genes (6). There are three types of negative regulators of the JAK/STAT signaling: suppressors of cytokine signaling (SOCS), inhibiting the phosphorylation of the receptor by JAK; protein inhibitors of activated STAT (PIAS), preventing the activity of STAT transcription factors; and protein tyrosine phosphatases (PTPs), removing phosphate groups from JAKs and STATs.

The activation of this pathway begins when specific cytokines, such as interleukins, interferons, or growth factors, bind to their corresponding cell surface receptors [27]. These receptors are often physically associated with JAKs, which possess tyrosine kinase activity. Upon cytokine binding, a conformational change induces the dimerization of the receptor molecules, leading to the transphosphorylation and activation of JAKs that are noncovalently attached to the receptors (Figure 1). Activated JAKs phosphorylate tyrosine residues at the cytoplasmic tail of the receptor, and this event, in turn, recruits the STAT protein family to the receptor by creating docking sites for STATs, which have Src homology 2 (SH2) domains. Once recruited to the receptor, STAT proteins are phosphorylated by JAKs at specific tyrosine residues, leading to their dimerization. These phosphorylated and dimerized STATs can now translocate into the nucleus, where they regulate the expression of their target genes (Figure 1).

### 2.2. JAKs and STATs

There are four types of JAK proteins, JAK 1, 2, 3, and tyrosine kinase 2 (TYK2), as well as seven types of STAT proteins, STAT 1, 2, 3, 4, 5A, 5B, and 6. Although their roles differ slightly, they share common structures. Each JAK protein consists of seven homology domains known as Janus homology (JH) domains [28]. These seven JH domains are divided into four functional domains. The kinase domain, corresponding to JH domain 1, is responsible for the kinase activity, providing the ATP binding site [29]. The pseudokinase domain, containing JH domain 2, shares structural similarity with the kinase domain but does not participate in kinase activity. Instead, this domain facilitates interactions between JAK and STAT molecules. The SH2 domain and the four-point one, ezrin, radixin, moesin (FERM) domain regulate protein–protein interactions, including the noncovalent attachment of JAK to cytokine receptors [14,30]. While the SH2 domain typically recognizes and binds to phosphotyrosine residues, there is a perspective that the SH2 domain in JAK differs from the standard SH2 domain because JAK can bind to cytokine receptors without relevant phosphotyrosine residues. Instead, it is speculated that the SH2 domain in JAK acts as a scaffold [31]. The FERM domain mediates protein interactions with membrane-associated proteins [31,32].

STAT proteins consist of several domains: N-terminal domain, coiled-coil domain, DNA-binding domain (DBD), linker domain, SH2 domain, and transcription activation domain (TAD) [14,29]. The N-terminal domain plays a crucial role in the dimerization and phosphorylation of STAT [31,33]. The coiled-coil domain is involved in nuclear import and export. The DBD facilitates binding to the target DNA sequence. The linker domain connects the DBD and the SH2 domain, providing structural support [34]. The SH2 domain of STATs recognizes the phosphotyrosine residue in the cytoplasmic tail of cytokine receptors, which is phosphorylated by JAK following ligand-mediated receptor dimerization (Figure 1). The binding of STATs to cytokine receptors through the interaction between the SH2 domain and phosphotyrosine residues renders STATs physically proximal to JAK. This proximity enables JAK to efficiently phosphorylate STAT, leading to dimer formation and nuclear import of the transcriptional factors [28,29,34]. Inside the nucleus, the activated STAT dimers interact with specific DNA sequences called STAT response elements (SREs) in the promoters or enhancers of target genes. This interaction triggers transcriptional activation, leading to the expression of genes that modulate essential cellular functions, including growth, differentiation, and homeostasis (Table 1).

### 2.3. Cytokines Activating the JAK/STAT Signaling Pathway

Various ligands trigger the initiation of the JAK/STAT signaling pathway, with cytokines being one of the most representative examples. Cytokines are small molecules secreted by a variety of cells that facilitate interactions and communication between cells [39]. They play a pivotal role in mediating a wide range of cellular functions, including cell growth, adhesion, differentiation, proliferation, and apoptosis, as well as the regulation of immunity and inflammatory responses. Cytokines have been intensively studied in the context of cancer as well [40]. Cytokines known to ignite the JAK/STAT signaling pathway encompass interleukins (IL), colony-stimulating factors, growth factors (GFs), and interferons (IFN) [37,41,42] (See Table 2).

The IL-2 family cytokines, including IL-2, IL-4, IL-7, IL-9, IL-15, and IL-21, engage their respective receptors, which consist of heterodimeric or heterotrimeric complexes composed of various cytokine-specific receptor chains. Upon ligand binding, these receptors undergo conformational changes that facilitate the recruitment and activation of JAK family members, primarily JAK1 and JAK3 [43,44]. These receptor-associated JAKs phosphorylate each other and the cytoplasmic tails of cytokine receptors, creating docking sites for STATs. Notably, the IL-2 family cytokines activate specific STAT proteins, such as STAT5 for IL-2, IL-15, and IL-21, and STAT6 for IL-4 [44,45,46]. This culminates in the transcriptional regulation of genes critical for cell survival, proliferation, differentiation, and immune responses [28,38].

The IL-6 family of cytokines, including IL-6, IL-11, IL-27, IL-31, and leukemia inhibitory factor (LIF), engages their cognate receptors, which generally consist of gp130 as a common receptor subunit, along with specific ligand-binding receptor chains [47,48]. IL-6 family proteins induce the activation of JAK1 and JAK2 primarily, as well as tyrosine kinase 2 (TYK2) for some members such as IL-6 and IL-11. Notably, the IL-6 family cytokines predominantly lead to the activation of STAT3 [23,49,50,51,52]. It is worth noting that certain members, such as IL-27, can also activate STAT1, influencing specific immune responses [53,54].

**Table 2:** Ligands triggering the activation of JAK/STAT signaling in HCC.

Ligand	JAK	STAT	Reference
IL-2 family (IL-2, 4, 7, 9, 15, 21)	JAK1, JAK3	STAT1, STAT3, STAT5, STAT6	[27,36,55,56,57]
IL-6 family (IL-6, 11, 27, 31, LIF)	JAK1, JAK2, TYK2	STAT1, STAT3	[27,36,55,56,58]
IL-10 family (IL-10, 19, 20, 22, 24, 26)	JAK1, TYK2	STAT1, STAT3, STAT5	[27,36,59,60,61]
IL-12 family (IL-12, 23)	JAK2	STAT3, STAT4	[36,55,56]
IL-17 family (IL-17A-F)	JAK2	STAT3	[62,63]
β common cytokine family (IL-3, 5, GM-CSF)	JAK2	STAT3, STAT5	[27,36,55,56]
Hematopoietic growth factors (EPO, G-CSF, TPO)	JAK2	STAT3, STAT5	[36,55,56,57]
Type1 IFN (α, β)	JAK1, TYK2	STAT1, STAT2	[36,37,55,56,58]
Type2 IFN (γ)	JAK1, JAK2	STAT1	[27,36,37,55,56,57,58]

The table lists cytokines, growth factors, and interferons that are predominantly enriched in HCC. It also provides information about the major JAKs and STATs affected by each type of ligand.

The IL-10 family includes IL-10 itself, as well as IL-19, IL-20, IL-22, IL-24, and IL-26. These cytokines engage specific receptor complexes, typically composed of receptor subunits such as IL-10R1, IL-10R2, and IL-22R1 [64]. Upon ligand binding, the receptor complex activates JAK1 and TYK2, in particular. The IL-10 family cytokines predominantly activate STAT3, in addition to STAT1 and STAT5 [65,66,67,68]. Similarly, the IL-12 family, consisting of IL-12 and IL-23, leads to the activation of JAK2. IL-12 predominantly signals through STAT4, while IL-23 induces STAT3 phosphorylation [69,70]. The IL-17 family cytokines, which include IL-17A, IL-17B, IL-17C, IL-17D, IL-17E (also known as IL-25), and IL-17F, trigger JAK/STAT signaling cascade through the activation of JAK2 and STAT3 [62,63].

The β common cytokine family includes cytokines such as IL-3, IL-5, and granulocyte-macrophage colony-stimulating factor (GM-CSF), which share a common β subunit (CD131) in their receptor complexes [71]. The ligand–receptor complex primarily activates JAK2. The β common cytokines mainly activate STAT5, which becomes phosphorylated, dimerizes, and translocates into the nucleus to drive gene expression [72]. Additionally, these cytokines can also induce activation of STAT3 to varying degrees [73,74].

Hematopoietic GFs, including erythropoietin (EPO), granulocyte colony-stimulating factor (G-CSF), and thrombopoietin (TPO), engage their respective receptors to initiate signal transduction cascades. These receptors typically consist of single or multiple subunits, with some sharing the common β subunit (CD131), and predominantly activate JAK2, leading to the activation of specific members of STATs [20,22,75,76,77,78,79]. For instance, EPO activates JAK2 and STAT5, while G-CSF triggers JAK2 activation and subsequent STAT3 phosphorylation, stimulating the proliferation and differentiation of granulocyte lineage cells. TPO, engaging JAK2, primarily activates STAT3, promoting the development and maturation of platelets.

Type 1 IFNs, including IFN-α and IFN-β, are produced in response to viral infections and engage a heterodimeric receptor complex composed of IFNAR1 and IFNAR2 subunits. Upon ligand binding, this receptor complex triggers the activation of JAK1 and TYK2. These JAKs primarily phosphorylate STAT1 and, to a lesser extent, STAT2 [80]. On the other hand, Type 2 IFN, mainly IFN-γ, engages a receptor complex consisting of IFNGR1 and IFNGR2 subunits. Ligand binding leads to the activation of JAK1 and JAK2, which phosphorylate STAT1, leading to the formation of homodimers or heterodimers with STAT3 [81].

### 2.4. Receptors and Negative Regulators of the JAK/STAT Signaling Pathway

Initiation of JAK/STAT signaling occurs when the aforementioned ligands, primarily cytokines, bind to transmembrane receptors. The types of transmembrane receptors involved in the JAK/STAT pathway include cytokine receptors, G-protein coupled receptors, and growth factor receptors [82]. Given the diverse range of ligands that induce JAK/STAT signaling transduction, there exists a multitude of receptors. These receptors can be categorized into Class I and Class II receptors [31,32,64].

Class I receptors feature conserved pairs of cysteines connected by disulfide bonds. This class of receptors is further divided into the glycoprotein 130 (gp130) family, the γ chain family, the β chain family, the single-chain family, receptor tyrosine kinases, and other interleukin receptors. Within the glycoprotein 130 (gp130) family, members include the IL-6R family (IL-6R, IL-11R, IL-12R, IL-23R), the G-CSF receptor, and the leukemia inhibitory factor receptor chain (LIFR). The interaction of gp130 with JAK1, JAK2, and TYK2 leads to the activation of STAT1, STAT3, and STAT5. Additionally, the γ chain family encompasses receptors for the IL-2 family (IL-2, IL-4, IL-7, IL-9, IL-15, and IL-21). Another subset of Class I receptors is the β chain family, which involves IL-3R, IL-5R, and the granulocyte-macrophage colony-stimulating factor (GM-CSF) receptor [83]. The single-chain family, characterized by homomeric receptors, includes receptors such as the thrombopoietin receptor (TPOR), erythropoietin receptor (EPOR), prolactin receptor (PRLR), and growth hormone receptor (GHR), linked with JAK2 and STAT5. On the other hand, Class II receptors are divided into antiviral and nonantiviral categories. The antiviral subset primarily engages with interferons (IFNs), while the nonantiviral subset includes receptors for IL-10 family cytokines such as IL-10, IL-20, IL-22, and IL-24. This comprehensive classification sheds light on the intricate landscape of JAK-STAT signaling receptors and their connections.

The JAK/STAT signaling pathway is crucial for transmitting signals from cell surface receptors, primarily those of cytokines, to the nucleus, where it regulates the expression of numerous genes involved in various cellular processes. To maintain appropriate cellular responses, the pathway requires tight regulation. In addition to the numerous activators of this signaling pathway, including various growth factors and cytokines, there are negative regulators that play a vital role in inhibiting the JAK/STAT pathway. There are three categories of negative regulators: suppressors of cytokine signaling (SOCS), protein inhibitors of activated STAT (PIAS), and protein tyrosine phosphatases (PTPs) (Figure 1).

The SOCS family encompasses eight distinct protein members: cytokine-inducible SH2 domain protein (CISH), SOCS1, SOCS2, SOCS3, SOCS4, SOCS5, SOCS6, and SOCS7. The SOCS proteins bind to the activation loop within the kinase domain of JAK or the cytoplasmic tail of the receptor. For instance, SOCS1 binds to activated JAKs, while SOCS3 binds to tyrosine residues on receptors. This binding efficiently blocks the phosphorylation of the receptor by JAKs [14]. Additionally, SOCS proteins function as E3 ubiquitin ligases and mediate ubiquitination-driven degradation of signaling components of the JAK/STAT pathway [84,85]. The PIAS family consists of four protein members: PIAS1, PIAS2 (PIASx), PIAS3, and PIAS4 (PIASy). These molecules have been shown to inhibit the JAK/STAT signaling pathway by preventing STAT transcription factors from binding to their target genes [29,33]. Each PIAS protein interacts with a specific type of STAT protein. For example, PIAS1 inhibits the binding of STAT1 to DNA [86]. The final group consists of protein tyrosine phosphatases (PTPs). Given the significance of phosphorylation within the JAK/STAT signaling pathway, removing phosphate groups from tyrosine residues significantly affects the transmission of the signal. The prominent proteins in this category are SH2 domain-containing phosphatase-1 (SHP-1), SH2 domain-containing phosphatase-2 (SHP-2), and CD45 [87]. SHP proteins are involved in dephosphorylating both JAKs and STATs, with SHP-2 specifically interacting with STAT1, STAT3, and STAT5 [87]. CD45 is responsible for removing phosphates that are attached to JAKs [29].

## 3. Roles of JAK/STAT Signaling in HCC

### 3.1. Dysregulation of the JAK/STAT Signaling Pathway in Human HCC

The JAK/STAT signaling pathway, activated by numerous cytokines and growth factors, serves as a central communication hub in cellular activities [14,88]. Components of the JAK/STAT signaling can also affect the expression of a plethora of genes via epigenetic modifications of chromatin. For example, JAK2 directly phosphorylates Tyr 41 on histone H3, which prevents the binding of heterochromatin protein 1alpha (HP1alpha) [89]. Aberrant activation of the JAK/STAT signaling pathway confers malignant properties on cancer cells by dysregulating cellular proliferation and survival, as well as enhancing stem cell properties and inflammatory responses. The JAK/STAT signaling also promotes invasion and metastasis of cancer cells, partly achieved through the activation of the EMT [90]. In addition, this pathway is involved in creating an immunosuppressive microenvironment [23,91].

**Figure 2:**
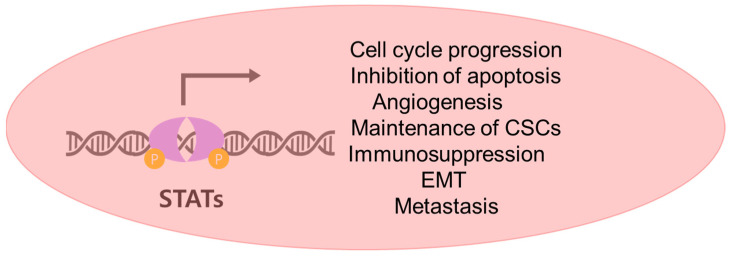
Roles of the JAK/STAT signaling in HCC.

The signals from the JAK/STAT pathway are carried out by the STAT transcription factors. In HCC, STAT3 is the major oncogenic player in various aspects of carcinogenesis. Genes that are transcriptionally activated by STAT3 in HCC include *CCND1* (encoding Cyclin D1), *BCL2*, *BIRC5* (encoding Survivin), *HIF-1α*, *VEGF*, *CD133* (encoding Prominin-1), *NANOG*, *IL-10*, *TWIST*, *MMP9*, etc. [92,93,94,95,96,97,98,99]. These target genes of STAT3 play critical roles in hepatocarcinogenesis by promoting cell cycle progression, inhibiting apoptosis, stimulating angiogenesis, maintaining CSCs, inducing immunosuppression, triggering EMT, and promoting metastasis (see Figure 2). Although controversial, other STAT proteins, notably STAT5, are also considered to exert similar tumor-promoting effects on HCC [88,100].

### 3.2. Frequencies of JAK and STAT Mutations Found in Human HCC

**Figure 3:**
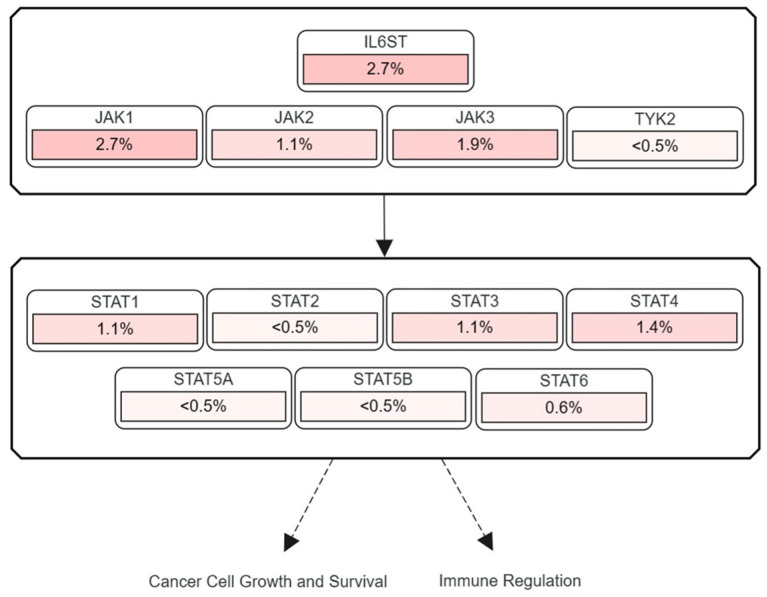
Frequencies of mutations in genes encoding JAK and STAT proteins in HCC. The highest mutation frequencies are observed in *IL6ST* (encoding the gp130 receptor) and *JAK1* among genes encoding major components of the JAK/STAT pathway.

Although human HCCs display dysregulation of the JAK/STAT signaling in approximately 50–60% of the cases, mutations in the components of the signaling cascade have been infrequently identified [101]. Activating mutations in *IL6ST* encoding the gp130 receptor can lead to phosphorylation of STAT3 in the absence of ligand binding [102]. Mutations in *IL6ST*, however, are found in less than 3% of HCC patients (Figure 3).

Various point mutations in *JAK1* have been observed in HCC, which lead to phosphorylation of JAK1 and STAT3 without the upstream stimulus [103]. For example, the mutation in codon 703 substituting isoleucine for the serine residue in JAK1 activates the JAK/STAT signaling pathway in vitro and in vivo without the ligand–receptor interaction [104]. However, mutations in *JAK1* are found in only 3% of HCC patients. Furthermore, mutations in the genes encoding other JAK proteins are found even less frequently than those in *JAK1*, undermining the contribution of JAK mutants to HCC (Figure 3). Activating mutations in STAT3 are predominantly found in the SH2 domain of STAT3, resulting in ligand-independent dimerization and phosphorylation of STAT3 [105,106]. Although STAT3 is phosphorylated in approximately 60% of patients with HCC, mutations in STAT3 are found in as low as 1% of human HCC. Mutations in other STAT proteins are found in less than 1% of patients with HCC (Figure 3).

### 3.3. Activation of the JAK/STAT Signaling via Downregulation of Negative Regulators

Although the frequencies of mutations in the major components of the JAK/STAT signaling pathway are found very low, abnormal activation of the JAK/STAT pathway is frequently found in HCC. Analysis of protein expression data from the Human Protein Atlas indicates that STAT3 exhibits the highest levels of expression within the JAK/STAT pathway proteins in HCC. Approximately 60% of HCC samples demonstrated activated STAT3 [91,107]. This suggests that apart from genetic mutations, alternative mechanisms should exist to promote the persistent activation of the JAK/STAT signaling pathway in HCC, such as overproduction of upstream ligands (e.g., cytokines and growth factors) or downregulation of negative regulators of the pathway.

Suppressor of cytokine signaling (SOCS) proteins function as inhibitors of the JAK/STAT signaling (Figure 1). SOCS hinder the ability of STAT proteins to bind with JAK and subsequently modulates the signaling pathways triggered by cytokines, thus suppressing the phosphorylation of STAT by JAK. SOCS can also suppress the JAK/STAT signaling by acting as ubiquitin ligases, leading to proteasomal degradation of JAK [90]. Hypermethylation and gene silencing of SOCS have been frequently observed in subsets of HCC, notably in hepatitis B virus-related HCC, with a frequency ranging from 40.9% to 67% [108,109]. Similarly, downregulation of other negative regulators such as PIA and SHP via promoter hypermethylation has been observed in HCC [110,111].

### 3.4. Overproduction of Inflammatory Cytokines Activating JAK/STAT Signaling in HCC

**Figure 4:**
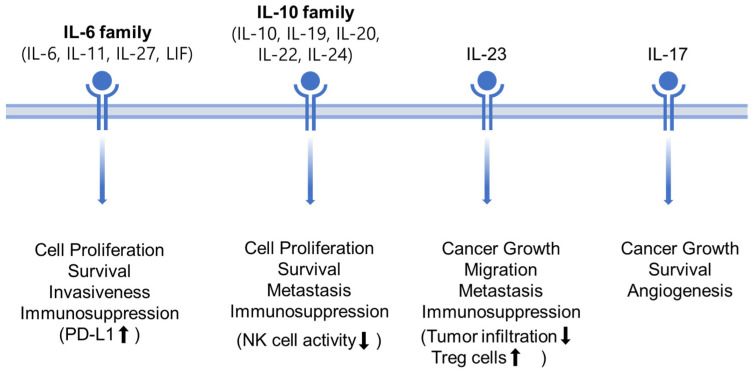
Roles of cytokines activating the JAK/STAT signaling in HCC. Upward and downward pointing arrows in parentheses indicate activation and suppression, respectively.

The livers of HCC patients are often infiltrated with different types of inflammatory cells that secrete various types of cytokines, growth factors, chemokines, and other molecules [50,112]. Dysregulated production of inflammatory cytokines is considered a major trigger for HCC carcinogenesis and tumor progression [113]. Among the numerous inflammatory cytokines associated with the activation of the JAK/STAT signaling in HCC, this review focuses on IL-6 family cytokines, IL-10 family cytokines, IL-23 as a member of IL-12 family cytokines, and IL-17, which play critical roles in the pathogenesis of HCC (Figure 4).

#### 3.4.1. IL-6 Family (IL-6, IL-11, IL-27, LIF)

IL-6 family cytokines, which activate the JAK/STAT signaling pathway, have been linked to the initiation and progression of HCC [50]. Both T cells and macrophages secrete IL-6 [114], and its expression plays a role in the growth, invasion, and progression of various cancer types, including HCC [115,116,117]. Elevated levels of interleukin-6 (IL-6) have been documented in the bloodstream across various hepatic disorders susceptible to the development of hepatocellular carcinoma (HCC) [118]. Elevated serum IL-6 levels have shown a positive correlation with an increased propensity for HCC development [119]. Moreover, individuals with HCC display elevated serum levels of both IL-6 and the IL-6 receptor [120]. Besides its oncogenic properties conferred on tumor cells through the JAK/STAT signaling pathway, IL-6 promotes PD-L1 expression in monocytes and macrophages in human HCC, contributing to immunosuppression in patients [114]. Similarly, IL-27 is significantly increased in HCC patients [50]. IL-27 plays a role in regulating inflammation within the tumor microenvironment and influences the proliferation, survival, and invasiveness of tumor cells. These effects are mediated through downstream signaling pathways, notably the JAK/STAT3 pathway [121]. Another IL-6 family cytokine, LIF, has also been observed to be significantly upregulated in HCC [122].

#### 3.4.2. IL-10 Family (IL-10, IL-19, IL-20, IL-22, IL-24)

Interleukin-10 (IL-10) family cytokines play a pivotal role in the progression of liver diseases. Beyond their diverse immunoregulatory functions, IL-10 influences susceptibility to HCV infection and hepatic fibrogenesis [123,124]. A study revealed higher IL-10 secretion in liver cancer cells compared to normal hepatocytes [125]. In terms of anti-inflammatory response, IL-10 binds to its receptor complex (IL10R1, IL10R2), which associates with JAK1 and TYK2, subsequently engaging STAT3 as a transcription factor [126]. IL-10 levels were found to be positively correlated with p-STAT3 levels. IL-10 serves as a critical negative regulator of NK cell activity. The release of IL-10 by HCC cells activates the STAT3 signaling pathway in NK cells, inhibiting their killing activity. This results in promotion of HCC recurrence and metastasis [127]. Inhibiting STAT3 in HCC cells led to decreased production of immunosuppressive IL-10 cytokines and reduced the number of regulatory T (Treg) cells, thereby alleviating their combined inhibitory effect on NK cells [128].

Despite sharing structural similarities and utilizing similar or partially identical receptors, different members of the IL-10 family exhibit distinct biological functions [129,130]. Interleukin-19 (IL-19) triggers its own expression (auto-induction) and the expression of other pro-inflammatory cytokines, leading to the activation of cell apoptosis. It specifically targets hepatocytes and stimulates the production of TNF-α and IL-10 [131]. IL-19 is also involved in liver disease-associated wound healing, serving as an initial step in processes such as fibrogenesis and cirrhosis, which subsequently promote compensatory proliferation of hepatocytes [132]. In primary murine hepatocytes, IL-19 binds to the IL-20R1/IL-20R2 heteroreceptor complex, activating STAT3 and triggering cell proliferation [133,134]. STAT3 plays a dominant role in negatively regulating the immune response, as well as governing cell growth, differentiation, apoptosis, and tumor development and metastasis [14].

IL-20 signals through its binding to either IL-22R/IL-20R2 or IL-20R1/IL-20R2. Both of these heterodimer receptor complexes activate the JAK/STAT pathway. Interestingly, the presence of constitutive IL-20R2 expression on hepatocytes alone is enough to activate STAT3 [135,136]. IL-20 plays a crucial role in the development of liver injury. In liver biopsies from patients with fibrosis, cirrhosis, and hepatocellular carcinoma, IL-20 levels were significantly elevated in hepatocytes and hepatic stellate cells compared to normal liver tissue from healthy individuals [137]. IL-20 shows a positive correlation with cyclin D1 expression in both HCC patients and human HCC cell lines [138]. Moreover, the treatment with an anti-IL-20 monoclonal antibody effectively hindered HCC cell proliferation, migration, and invasion in vitro and demonstrated the suppression of liver tumor growth in vivo [139].

#### 3.4.3. IL-23 and IL-17

IL-23 belongs to the IL-12 family of cytokines and acts as a crucial molecular link between tumor-promoting pro-inflammatory processes and the impairment of adaptive immune surveillance [140]. Upon binding to its receptor IL-23R, JAK2 and TYK2 are activated, leading to subsequent STAT3 phosphorylation [141]. In the context of HCC, IL-23 plays a pivotal role in promoting cancer growth, progression, and metastasis. It also diminishes CD8+ cell infiltration in tumors while enhancing the immunosuppressive function of Treg cells. Moreover, elevated IL-23 expression levels in HCC are correlated with advanced TNM stage and metastasis [142].

Interleukin-17 (IL-17), which exhibits increased expression in HCC, can facilitate the invasion and migration of HCC cells [62,143]. The tumor-promoting effects of IL-17 and its receptor IL-17R are achieved through direct influence on tumor cells via the IL-6/JAK2/STAT3-related pathway. Aberrantly activated STAT3 in HCC cells, in turn, leads to the upregulation of IL-17 [144]. IL-17 plays a vital role in attracting neutrophils to the peritumoral stroma of HCC tissues and promoting pro-angiogenic activity in HCC cells [145]. The interaction between IL-17 and IFN-γ has significant effects on HCC cell apoptosis and growth in both in vitro and in vivo settings. IL-17 counteracts the antitumor effects of IFN-γ in HCC cells, thereby promoting HCC development [63].

## 4. In Vitro and In Vivo Studies Targeting the JAK/STAT Signaling in HCC

As discussed above, the upregulation of JAK/STAT signaling significantly contributes to the increased proliferation, migration, and invasion of HCC cells. Furthermore, this up-regulation leads to enhanced cell survival and metastasis. Given that the JAK/STAT signaling pathway exerts strong tumor-promoting effects on HCC, it is reasonable to consider inhibiting the pathway for the treatment of HCC. In vitro investigations have consistently indicated that inhibiting JAK/STAT activity results in increased apoptosis in HCC cells, thereby reducing cell survival. Similarly, in vivo preclinical studies have also convincingly demonstrated that blocking JAK/STAT activity can effectively impede tumor growth or even delay the progression of tumors.

### 4.1. In Vitro Studies Targeting the JAK/STAT Signaling Pathway in HCC Cells

The SOCS proteins act as endogenous inhibitors of the JAK/STAT signaling pathway. Frequent observations of hypermethylation or histone modification in the promoter regions of SOCS have been made in HCC [146,147]. These events result in the loss of normal SOCS function, contributing to the activation of the JAK/STAT signaling pathway and the subsequent development of HCC. The epigenetic suppression of SOCS in HCC can be countered by inhibiting DNA methyltransferase (DNMT) using Decitabine (5-aza-2′-deoxycytidine or 5-Aza-CdR) and inhibiting histone deacetylase (HDAC) using Vorinostat (suberoylanilide hydroxamic acid or SAHA). Treatment with these agents elevates the expression levels of SOCS, leading to JAK/STAT pathway inhibition. Upon treatment with these agents, human HCC cells (HLE, LCL-PI 11) exhibited increased expression of SOCS1 and SOCS3, resulting in reduced activation of JAK2 and STAT3. Consequently, cell growth was hindered, and apoptosis was enhanced [148].

JAK1 was identified as a target of MicroRNA 30e (miR-30e). Transfection of HCC cells (HepG2 and Huh-7) with miR-30e led to decreases in phosphorylated levels of JAK1 and STAT3. Additionally, overexpression of miR-30e resulted in reduced cell proliferation, migration, and invasion, along with increased apoptosis in the HCC cells [149]. Pharmacological inhibition of JAK1 was also attempted using baricitinib, a small-molecule and selective inhibitor of JAK1 [150]. Treating HepG2 and Huh-7 cells with baricitinib downregulated phosphorylation of JAK1 and STAT3, resulting in reduced nuclear translocation of STAT3. Baricitinib administration led to decreased cell growth and increased apoptosis in the HCC cells [151].

Etomidate inhibits phosphorylation and activation of JAK2 by competing with ATP for binding to the ATP-binding pocket of JAK2. Application of etomidate to HCC cells (HepG2 and Huh-7) suppressed phosphorylation of JAK2 and STAT3, resulting in the inhibition of cell proliferation, migration, and invasion, coupled with enhanced apoptosis [152]. Similarly, momelotinib, another small-molecule JAK2 inhibitor, suppressed phosphorylated JAK2 and STAT3 levels upon treatment of HCC cells (SNU-378 and SNU-475), subsequently leading to reduced cell viability, migration, and invasion [153].

Targeting STAT proteins also exerts similar anticancer effects in HCC cells. Pravastatin, a nonpeptide small molecule, effectively impedes STAT1 phosphorylation upon the activation by cytokine-induced pathways. Notably, pravastatin effectively dampened the phosphorylation of STAT1 induced by IFN-γ [154]. The proliferation of HepG2 and Huh-7 cells was significantly decreased following the treatment with pravastatin [155].

Napabucasin (BBI608) is a novel small-molecule inhibitor of STAT3. It has been shown to impair cancer stemness in various types of cancers, such as colorectal, pancreatic, and prostate cancers [156,157]. In a variety of HCC cells, including Huh-7, Hepa1-6, and HepG2, treatment with napabucasin led to decreases in cell viability and cell growth. Importantly, the treatment resulted in the downregulation of pSTAT3 and stemness markers such as Nanog, CD133, Klf4, Oct4, and Sox2 [158]. In another study, tumors induced by activated beta-catenin and mTOR signaling were highly susceptible to napabucasin, which strongly inhibited the proliferation of the tumor cells [159].

### 4.2. Preclinical Animal Studies Targeting the JAK/STAT Signaling Pathway in HCC

Studies have demonstrated that miR-515-5p directly binds to the 3′-untranslated region (3′-UTR) of interleukin 6 (IL-6). Treatment of HCC cells (HepG2) with miR-515-5p suppressed the JAK/STAT signaling pathway along with reduced IL-6 expression. Overexpression of miR-515-5p inhibited migration and invasion of HepG2 cells that were transplanted into nude mice [160]. Similarly, in xenograft mice transplanted with HepG2 cells, treatment with etomidate, an inhibitor of JAK2, led to the inhibition of tumor growth and subsequent increases in animal survival (Table 3) [152].

SOCS3 is a suppressor of the JAK/STAT signaling. A long noncoding RNA known as C1QTNF1 antisense RNA 1 (C1QTNF1-AS1) maintains SOCS3 activity by binding with miR-221-3p, which downregulates SOCS3. In nude mice transplanted with HCC cells (HepG2 or Huh-7), ectopic expression of C1QTNF1-AS1 led to suppression of HCC tumor growths in vivo which was accompanied by a decrease in the expression of phosphorylated STAT3, as well as an increase in SOCS3 expression [161]. JAK2 and STAT3 are downstream signaling molecules of EphB4 receptor. In HCC cells, inhibition of EphB4 by the treatment with cantharidin led to suppression of JAK2 and STAT3 activities. In xenograft murine HCC models, treatment with cantharidin suppressed tumor growth in the mice [162]. Nitidine chloride is a small-molecule inhibitor that blocks the phosphorylation and activation of JAK1 and STAT3. When nitidine chloride was administered to nude mice transplanted with HepG2 cells, there was a decrease in the expression of phosphorylated JAK1 and STAT3 in the tumors, which resulted in an increase in apoptotic cells and a decrease in proliferating cells. These effects ultimately led to the inhibition of tumor growth and a reduction in tumor weight, thus confirming the anticancer efficacy of nitidine chloride [163].

Dehydrocrenatidine is a potent JAK family kinase inhibitor. HCC cells treated with dehydrocrenatidine exhibited a significant suppression in the phosphorylated level of JAK2. HepG2-xenograft mice treated with dehydrocrenatidine showed a significant reduction in tumor volume, leading to increases in animal survival. Of note, the treatment led to significantly reduced EMT and CSC phenotypes in HCC [164]. An azaspirane derivative, CIMO is an inhibitor of JAK/STAT signaling pathway by targeting JAK2 and JAK3. In HCC cell lines such as HepG2 and Hep3B, CIMO inhibited JAKs and STAT3 phosphorylation. In orthotopic murine xenograft models of HCC, treatment with CIMO at a dose of 10 mg per kg body weight led to a significant decrease in tumor growth [165].

Additionally, attempts to inhibit STAT3 have been made in HCC murine models. Mice transplanted with Hepa1-6 cells were intraperitoneally injected with napabucasin, a STAT3 inhibitor, at a dose of 20 mg per kilogram of body weight, or vehicle. Napabucasin treatment significantly reduced tumor volumes compared to the control group. Notably, approximately 25% of mice showed tumor regression when examined 21 days after administration [158]. OPB-31121 is another potent inhibitor of STAT3 phosphorylation that has demonstrated significant growth inhibition in various hematopoietic malignant cells [166]. Immunodeficient mice transplanted with Huh-7 or HepG2 cells were orally administered OPB-31121 at a daily dose of 60 mg per kg of body weight for 14 days. Treatment with OPB-31121 significantly inhibited HCC growth in the murine models [167].

In line with the results from xenograft models of HCC, chemically induced autochthonous HCC models also showed inhibition of the JAK/STAT signaling suppressed HCC development. For example, rats harboring autochthonous HCC were treated with pravastatin, an inhibitor of STAT1 phosphorylation. The treatment led to significantly smaller tumor sizes in the livers. Immunohistochemistry studies revealed that the level of cellular proliferation in HCC from rats treated with pravastatin was significantly diminished compared to that from untreated mice [168].

## 5. Clinical Studies Targeting the JAK/STAT Signaling Pathway in HCC

The significant role of the JAK/STAT signaling pathway in HCC suggests that targeted inhibition of this pathway could hold therapeutic potential for treating patients with HCC [90]. Various types of JAK/STAT inhibitors are currently under investigation for their clinical relevance in HCC (Table 4). Itacitinib, an oral selective inhibitor of JAK1, has demonstrated no serious treatment-related adverse events [169,170]. A single-arm Phase Ib study is currently underway to evaluate the effect of itacitinib on 25 patients with advanced HCC (NCT04358185).

Considering the tumor-suppressive effects of pravastatin in preclinical animal models of HCC [168], the STAT1 inhibitor was assessed for its anticancer effects in clinical studies (NCT01418729, NCT01903694, and NCT01075555). In a Phase III clinical trial, patients with HCC received either sorafenib alone, a standard therapeutic agent for HCC, or sorafenib plus pravastatin. However, the combination of pravastatin with sorafenib failed to provide additional benefits to patients, as the mean overall survivals were not significantly different between the sorafenib alone and sorafenib plus pravastatin groups (10.7 months and 10.5 months, respectively) [171].

Danvatirsen (also known as AZD9150) is an antisense oligonucleotide that binds to STAT3 mRNA, thereby inhibiting the translation of STAT3 mRNA [172]. In patients with lymphoma and lung cancer, danvatirsen exhibited antitumor activity [173]. A Phase I trial assessed its safety and antitumor activity in patients with advanced or metastatic HCC (NCT01839604). Although the drug appeared relatively safe, it did not consistently show an antitumor effect [174].

In vitro and in vivo animal studies have demonstrated that napabucasin has a strong antitumor effect on HCC, as the STAT3 inhibitor suppresses cell viability and proliferation, as well as downregulating expression of stemness genes [158,159]. Based on promising preclinical results, a clinical trial evaluated the co-administration of sorafenib and napabucasin for potential benefits in advanced HCC patients over sorafenib treatment alone. However, a Phase I/II clinical trial revealed that co-administration of sorafenib and napabucasin achieved no improvement in overall response rates in HCC patients, compared to the treatment with sorafenib alone (NCT02279719).

Similarly, due to its effective suppression of tumor growth in murine HCC models [167], OPB-31121, a potent STAT3 inhibitor, was administered to 23 patients with progressive HCC at varying doses. To evaluate its antitumor efficacy, overall responses were assessed (NCT01406574). From the clinical trial, a marginal effect was observed in only six out of the twenty-three patients for a maximum of 8 weeks [167].

It is important to note that while inhibition of the JAK/STAT signaling pathway has shown promise in preclinical studies, its efficacy in patients with advanced HCC remains uncertain. Current clinical trials focused on this strategy are still in their early stages, and more studies are needed to better assess the effectiveness of targeting this signaling pathway. In summary, the ongoing clinical studies targeting the JAK/STAT pathway emphasize the complexity of this approach in HCC treatment and underscore the necessity for further investigation to identify more effective treatment strategies.

## 6. Perspectives and Conclusions

In the realm of clinical translation, it is noteworthy that while targeting the JAK/STAT pathway in hepatocellular carcinoma (HCC) holds great promise, ongoing clinical trials focused on this strategy are still in their infancy. Despite the well-established significance of aberrant JAK/STAT signaling in HCC pathogenesis, along with preclinical observations of the anticancer effects of targeting the pathway in HCC, the journey toward clinical implementation requires a more systematic approach and refinement. Notably, adverse effects routinely observed in administering anticancer agents are particularly problematic in patients with HCC, given their pre-existing liver dysfunction due to sustained ailments from underlying chronic hepatitis and cirrhosis [1,2].

To explain the failure of therapeutic drugs that demonstrated antitumor effects in preclinical models of HCC to achieve effectiveness in clinical studies, several complex factors must be considered. In vitro and animal models of HCC do not perfectly mimic the human tumor microenvironment [175]. Factors such as immune response, blood supply, and interactions with neighboring tissues significantly influence HCC in humans [176]. Preclinical models might not accurately capture these aspects, leading to differences in drug response [177]. Additionally, human HCCs are genetically and molecularly diverse [178,179]. Genetic mutations, gene expression patterns, and signaling pathways can vary significantly among human patients. It is also noteworthy that animals and humans metabolize drugs differently due to varying pharmacokinetics between species. Thus, drugs that are effectively metabolized in animals to fully function as anticancer agents might not exhibit the desired effects on cancer in human due to imperfect metabolism of the drugs in the human body.

While current clinical studies targeting the JAK/STAT signaling pathway might not be yielding optimistic results, it is crucial to point out that these studies are still in the early stages. As researchers navigate the complexities of this pathway and its clinical translation, it is imperative that these efforts be met with the clinical successes they deserve. By shedding light on the underlying mechanisms driving JAK/STAT pathway activation and its contributions to HCC pathophysiology, we aim to pave the way for the development of novel JAK/STAT targeted therapies that hold promise in effectively combating this aggressive malignancy. Further, it is noteworthy that combining JAK/STAT targeted therapy with immunotherapy might hold promise, as both approaches involve targeting dysregulation of inflammatory and immune responses in the tumor microenvironment.

As an emerging therapeutic paradigm, the immune checkpoint has garnered attention as an attractive target for immunotherapy [180,181]. The rationale underpinning the immune checkpoint inhibition derives from the observation that the expression of programmed death-ligand 1 (PD-L1) in cancer cells allows them to evade immune surveillance via interaction with programmed death-1 (PD-1) receptors located on the surfaces of activated T cells, B cells, natural killer cells, and myeloid cells [182]. To maximize the effectiveness of immunotherapy in HCC, it is imperative to identify and develop new targets for immune modulators, thereby augmenting the efficacy of immune checkpoint inhibitors (ICIs). For instance, targeting Toll-like receptor 4 (TLR4) holds potential, given its significant role in orchestrating the immune response against HCC cells [183]. Similarly, activation of the NLRP3 inflammasome has demonstrated the ability to suppress HCC growth in murine xenograft models [184]. Hence, the combination of ICIs with TLR4 inhibitors or NLRP3 activators is poised to emerge as a promising immunotherapeutic approach for HCC. Furthermore, biomarkers are needed for predicting the response to immunotherapy and prognosis in HCC patients. In this context, Wu et al. recently reported the development of an immune-related gene prognostic index comprising nine immune-related genes [185].

Despite initial enthusiasm, targeting immune checkpoints in isolation has failed to demonstrate sustained therapeutic efficacy in HCC [180,182]. Consequently, therapeutic strategies have emerged that involve combining ICIs with other precisely targeted therapeutics [186,187]. In line with this concept, clinical trials have assessed the effectiveness of combinations involving anti-PD-1/PD-L1 antibodies with agents such as bevacizumab (targeting vascular endothelial growth factor-A), RTK inhibitors, or anti-CTLA-4 antibodies. This combinational therapeutic approach is currently regarded as the most efficacious modality for treating advanced HCC [186,188]. It is also intriguing to consider the potential effectiveness of combining JAK/STAT targeting with ICIs in patients with HCC.

## Figures and Tables

**Table 1:** Roles of JAK and STAT proteins.

Protein	Role	Reference
JAK1	Promotion of inflammatory response Antiviral effect Regulation of neurological and lymphocytic function	[14,28,35]
JAK2	Regulation of hematopoietic function	[14,28]
JAK3	Regulation of lymphocyte production and function	[14,28]
TYK2	Regulation of T cell response and microbial response	[14,28]
STAT1	Regulation of cell growth, differentiation, and apoptosis Inhibition of viral and bacterial infection	[14,18,25,28,34,36,37,38]
STAT2	Antiviral effect Immune regulation Promotion of tumorigenesis	[14,18,25,28,34,36,37,38]
STAT3	Regulation of immune system Progression of cell cycle Inhibition of apoptosis Promotion of tumorigenesis Maintenance of cancer stem cells (CSCs) Regulation of hepatic glucogenesis	[14,16,18,25,28,34,36,37,38]
STAT4	Regulation of immune system Regulation of T cell development and function	[14,18,25,28,34,36,37,38]
STAT5	Regulation of cell growth, differentiation, and apoptosis Regulation of tumor immunity Tumor progression	[14,18,25,28,34,36,37,38]
STAT6	Regulation of lymphocytes Expression of MHCs and immunoglobulins	[14,18,25,28,34,36,37,38]

**Table 3:** HCC suppression in xenograft models by inhibiting the JAK/STAT signaling pathway.

Agent	Target Molecule (Inhibited Molecule)	HCC Cell Line Transplanted	Phenotype	Reference
miR-515-5p	IL-6 (STAT3)	HepG2	Inhibited migration and invasion of HCC cells	[160]
Etomidate	JAK2	HepG2	Inhibition of tumor growth Increase in animal survival	[152]
C1QTNF1-AS1	miR-221-3p (STAT3)	HepG2, Huh-7	Reduced tumor volumes	[161]
Cantharidin	EphB4 receptor (JAK2, STAT3)	SMMC-7721	Reduced tumor growth	[162]
Nitidine chloride	JAK1, STAT3	HepG2	Reduced tumor volumes	[163]
Dehydrocrenatidine	JAK2	HepG2	Reduced invasion of HCC Inhibition of CSC phenotypes	[164]
CIMO	JAK1, JAK2, STAT3	Huh-7	Reduced tumor growth	[165]
Napabucasin	STAT3	Hepa1-6	Reduced tumor volumes Tumor regression in 25% mice	[158]
OPB-31121	STAT3	Huh-7, HepG2	Reduced tumor growth	[166]

**Table 4:** Clinical studies targeting the JAK/STAT signaling pathway in HCC.

Drug	Target	NCT Number	Phase	Status	Clinical Outcomes
Itacitinib	JAK1	04358185	Ib	In progress,	Not yet determined
Pravastatin	STAT1	01075555	III	Completed (September 2015)	MOS 10.7 m vs. 10.5 m (sorafenib vs. pravastatin + sorafenib)
Danvatirsen	STAT3	01839604	I	Completed (February 2015)	Limited antitumor activity.
Napabucasin	STAT3	02279719	I/II	Completed (October 2019)	No significant difference in overall response rates between sorafenib alone and napabucasin + sorafenib groups
OPB-31121	STAT3	01406574	I/II	Completed (March 2014)	Limited antitumor effects and low overall responses.

MOS, mean overall survival.

## Data Availability

Not applicable.
